# Association between electronic cigarette use and metabolic syndrome in the Korean general population: A nationwide population-based study

**DOI:** 10.1371/journal.pone.0237983

**Published:** 2020-08-21

**Authors:** Taeyun Kim, Hyunji Choi, Jihun Kang, Jehun Kim

**Affiliations:** 1 Division of Pulmonology, Department of Internal Medicine, The Armed Forces Goyang Hospital, Goyang-si, South Korea; 2 Department of Laboratory Medicine, Kosin University Gospel Hospital, Busan, South Korea; 3 Department of Family Medicine, Kosin University Gospel Hospital, Busan, South Korea; 4 Division of Pulmonology, Department of Internal Medicine, Kosin University Gospel Hospital, Busan, South Korea; University of Calfornia San Francisco, UNITED STATES

## Abstract

**Objectives:**

Although smoking is known to have a negative impact in patients with metabolic syndrome (MetS), only a few studies have examined the association between electronic cigarette (e-cig) use and MetS.

**Methods:**

Among 22,948 participants in the 6th Korea National Health and Nutrition Examination Survey, 14,738 (13,459 [91.3%] never, 954 [6.5%] ever, and 325 [2.2%] current e-cig users) were selected. The relationship between e-cig exposure and MetS (based on the National Cholesterol Education Program Adult Treatment Panel [NCEP-ATP] III criteria) was evaluated using a multivariable logistic regression analysis. An unweighted analysis was performed to evaluate this association without a sampling weight. A subgroup analysis was performed among active smokers to compare dual users with never e-cig users.

**Results:**

Among current e-cig users, 85.0% were dual users, 12.7% were former cigarette users, and 2.2% were only e-cig users. After adjustment for covariates, abdominal obesity and hypertriglyceridemia were significantly associated with current e-cig exposure (odds ratio [OR]: 1.88, 95% confidence interval [CI]: 1.41–2.50 and OR: 1.32, 95% CI: 1.00–1.74 respectively [compared with the never e-cig users group]). Compared with never e-cig users, current e-cig users showed an OR of 1.27 (95% CI: 0.96–1.70, *P*_trend_ = 0.01) for MetS. In the unweighted analysis, the OR for MetS in current e-cig users was 1.40 (95% CI: 1.08–1.81, *P*_trend_ <0.01). Compared with never e-cig users, dual users showed a higher OR for abdominal obesity (OR: 1.71, 95% CI: 1.25–2.34, *P*_trend_ <0.001).

**Conclusions:**

Current e-cig exposure was associated with an increased risk of MetS. Dual use of e-cigs and cigarettes was associated with abdominal obesity. Further longitudinal studies and better assessment of e-cig use and type are needed to clarify this relationship.

## Introduction

Since their introduction in 2007, the popularity of electronic cigarettes (e-cigs) has steadily increased among young populations, with 27.5% of high school students and 10.5% of middle school students reporting the use of e-cigs in 2019 [1]. Some adolescents and adults started using e-cigs alone, but the dual use of e-cigs and cigarettes is increasing [2]. In France, more than 80% of current e-cig users were dual users, and low income levels and unemployment were associated with high a probability of dual use [3].

As the e-cig-consuming population increases, various adverse health effects have been reported. E-cigs contain several chemical components; however, the actual composition of this product is not well known and there is a significant gap in the data on their health effects. In a crossover single-blind study conducted in 40 healthy individuals, several markers of oxidative stress were not different between e-cig users and traditional cigarette users [4]. Kaur et al. summarized the prolonged effects of e-cig consumption and concluded that long-term e-cig exposure may result in systemic inflammation [5].

Metabolic syndrome (MetS) is a term encompassing various metabolic statuses and adult diseases such as hypertriglyceridemia, low high-density lipoprotein (HDL) cholesterol levels, hypertension, diabetes mellitus, and central obesity [6]. Considering that previous studies have shown an association between MetS and cigarette use, smoking is an important risk factor for MetS [7]. Although evidence on the health-related adverse effects of cigarette use on MetS has been reported, to the best of our knowledge, only a few studies have evaluated the relationship between e-cig exposure and MetS. Since many cigarette users concurrently consume e-cigs [8], and since the chemical components of e-cigs are similar to those of cigarettes [9], it is necessary to measure the effect of e-cig use on MetS.

The South Korean government strongly recommended the suspension of the use of liquid-type e-cigs in October 2019. The results of our analysis on the relationship between e-cig use and metabolic variables can serve as a basis for the development and implementation of policies. We hypothesized that e-cig use may be associated with an increased risk for MetS and evaluated the association between e-cig use and MetS in the Korean general population using a nationwide representative sample.

## Materials and methods

### Study subjects

This cross-sectional study used data from the 6th Korea National Health and Nutrition Examination Survey (KNHANES) from 2013 to 2015. The KNHANES is an annual, nationally representative, population-based survey organized by the Korea Centers for Disease Control and Prevention. Briefly, the survey is designed using a stratified multistage probability sampling method to represent non-institutionalized Korean citizens and consists of health interviews, health examinations, and nutrition surveys. The detailed data profiles have been described previously [10]. The 6th KNHANES assessed 29,321 citizens of South Korea, of which 22,948 responded, with a response rate of 78.3%. The present study excluded participants (aged ≥19 years) with missing data regarding e-cig and cigarette use, MetS components, socio-economic status, and behavior patterns. Finally, 14,738 individuals were included in the analyses.

### Ethical approval

The study protocol was approved by the Institutional Review Board of the Kosin University Gospel Hospital (no. 2020-06-022), and the study was conducted according to the Declaration of Helsinki. All study procedures were performed in accordance with the Strengthening the Reporting of Observational studies in Epidemiology guidelines. Written informed consent was obtained from all individuals before participation in the survey.

### Data measurement

MetS was defined based on the modified Third National Cholesterol Education Program Expert Panel on Detection, Evaluation, and Treatment of High Blood Cholesterol in Adults (NCEP-ATP III) criteria [11] and the abdominal obesity criteria from the Korean Society for the Study of Obesity [12]. MetS was diagnosed when three or more of the following criteria were met: (a) waist circumference (WC) of ≥90 cm in men and ≥85 cm in women, (b) triglyceride (TG) concentration of ≥150 mg/dL, (b) HDL-cholesterol concentration of <40 mg/dL in men and <50 mg/dL in women, (d) blood pressure (BP) ≥130/85 mmHg or the current use of antihypertensive medication uses, and (e) fasting glucose concentration of ≥100 mg/dL or the current use of anti-diabetic medications. Blood samples were drawn by trained medical technicians and transported to the central NEODIN Medical Institute (Seoul, South Korea). Serum TG levels (mg/dL) were measured using an enzymatic method (Hitachi Automatic Analyzer 7600–210, Hitachi, Japan), HDL-cholesterol levels (mg/dL) were measured using a homogeneous enzymatic colorimetric method (Hitachi Automatic Analyzer 7600–210, Hitachi, Japan), and fasting glucose levels (mg/dL) were measured using a hexokinase ultraviolet method (Hitachi Automatic Analyzer 7600–210, Hitachi, Japan).

Data on e-cig use were gathered using a self-reported questionnaire, which included the following items: (a) “Have you ever used an e-cig in your lifetime?” and (b) “Have you used e-cigs within the past 30 days?” Participants who responded “no” to both questions were classified as never e-cig users. Participants who responded “yes” to the first question and “no” to the second question were classified as ever e-cig users. Participants who responded “yes” to both questions were classified as current e-cig users. Data on cigarette use were obtained based on the World Health Organization classification: a current smoker was defined as anyone who had smoked more than 100 cigarettes in their lifetime and smoked currently; a former smoker was defined as anyone who had smoked more than 100 cigarettes in the past and did not smoke currently; and a never smoker was defined as anyone who had ever smoked less than 100 cigarettes and did not smoke currently.

Information on alcohol consumption and physical activity were gathered from self-administered questionnaires or face-to-face interviews. High-risk alcohol consumption was defined as seven (60 g alcohol) or more drinks for men and five (40 g alcohol) or more drinks for women on a single occasion [13]. The frequency of high-risk alcohol consumption was classified as more than once and less than once per week. Adequate physical activity was defined as (a) at least 150–300 minutes of moderate-intensity physical activity per week, (b) 75–150 minutes of vigorous-intensity physical activity per week, or (c) an equivalent combination of moderate- and vigorous-intensity aerobic activities.

Body mass index (BMI) was calculated as body weight (kg) divided by height in meter squared (m^2^). Height and weight were measured to the nearest 0.1 cm and 0.1 kg, respectively. BMI was categorized based on the Korean Society for the Study of Obesity guidelines [12]; underweight (<18.5 kg/m^2^), normal (18.5–22.9 kg/m^2^), pre-obese (23–24.9 kg/m^2^), and obese (≥25 kg/m^2^).

### Statistical analysis

Since the KNHANES was designed using a multistage clustered probability sampling method to obtain a nationally representative sample of non-institutionalized Korean citizens, all statistical analyses applied a complex survey design and sampling weight.

The general characteristics of the study participants were categorized according to the status of e-cig use (never, ever, and current). Comparisons between e-cig use groups were performed using a one-way analysis of variance for normally distributed continuous variables and chi-square tests for categorical variables. Subsequently, a post-hoc analysis using Bonferroni correction was performed for multiple comparisons. *P*-values <0.017 were considered significant in the post-hoc analysis.

The associations between e-cig use and MetS components were evaluated using a multivariable logistic regression analysis with stepwise adjustment for covariates. Model 1 was adjusted for age and sex. Model 2 was adjusted for age, sex, and cigarette use. Model 3 was adjusted for age, sex, cigarette use, and alcohol consumption. Model 4 was adjusted for age, sex, cigarette use, alcohol consumption, physical activity, household income, and education level.

A multivariable logistic model was used to evaluate the association between e-cig use and MetS with stepwise adjustment for the above-mentioned covariates. The associations between e-cig use and MetS components were presented as odds ratios (ORs) with 95% confidence intervals (CIs). An unweighted analysis was also performed to determine whether analyses without sample weighting and clustering altered the associations observed in the weighted analysis. A subgroup analysis of active smokers was also conducted to minimize the effects of traditional cigarette use.

All statistical analyses were performed using IBM SPSS Statistics for Windows, version 25.0 (IBM Corp., Armonk, New York). *P*-values <0.05 were considered significant.

## Results

Table 1 presents the general characteristics of the participants according to the status of e-cig use. Among current e-cig users, 85.0% were dual users, 12.7% were former smokers, and 2.2% were only e-cig users. WC was greater in male current e-cig users than in male never e-cig users. TG levels were the highest in current e-cig users, followed by ever and never e-cig users. Diastolic BP was lower in never e-cig users than in ever and current e-cig users.

**Table 1:** Characteristics of the study participants according to electronic cigarette exposure (N = 14,738).

	Never users (n = 13,459)	Ever users (n = 954)	Current users (n = 325)	*P*-value
**Age (years)**	47.6 (0.2)	36.5 (0.4)	35.8 (0.6)	<0.001*,†
**Male**	51.1% (0.5)	89.9% (1.0)	88.2% (1.8)	<0.001*,†
**Smoking status**				<0.001*,†
Never	60.8% (0.5)	2.3% (0.5)	2.2% (0.8)	
Former	20.2% (0.4)	18.8% (1.5)	12.7% (2.0)	
Current	19.0% (0.5)	78.9% (1.6)	85.0% (2.2)	
**High-risk alcohol consumption**^a)^				<0.001*,†
≥1/week	52.9% (0.6)	54.9% (1.8)	56.2% (3.0)	
<1/week	47.1% (0.6)	45.1% (1.8)	43.8% (3.0)	
**Physical activity**^b)^				0.33
Yes	52.9% (0.6)	54.9% (1.8)	56.2% (3.0)	
No	47.1% (0.6)	45.1% (1.8)	43.8% (3.0)	
**Household income**				<0.01*,†
Lowest	11.8% (0.5)	8.7% (1.0)	7.3% (1.6)	
Lower middle	22.9% (0.6)	22.2% (1.5)	20.1% (2.5)	
Higher middle	30.9% (0.7)	35.4% (1.8)	39.2% (3.4)	
Highest	34.4% (0.9)	33.7% (1.7)	33.4% (3.2)	
**Educational level**				<0.001*,†
Middle school or lower	18.0% (0.5)	5.9% (0.8)	4.5% (1.1)	
High school	27.9% (0.6)	28.2% (1.6)	31.1% (2.8)	
College or more	54.1% (0.8)	65.9% (1.7)	64.4% (2.9)	
**Body mass index**				<0.001*,†
<18.5 kg/m^2^	4.3% (0.2)	4.1% (0.8)	5.4% (1.6)	
18.5–22.9 kg/m^2^	40.6% (0.5)	29.9% (1.6)	29.9% (2.7)	
23.0–24.9 kg/m^2^	22.9% (0.4)	23.7% (1.5)	19.0% (2.3)	
25.0≥ kg/m^2^	32.2% (0.5)	42.2% (1.9)	45.8% (3.2)	
**Metabolic variables**				
WC (cm)				
Men	85.5 (0.1)	86.1 (0.4)	87.4 (0.7)	0.01†
Women	77.2 (0.2)	74.8 (0.9)	76.0 (1.7)	0.03*
Triglyceride (mg/dL)	136.4 (1.5)	173.1 (4.5)	185.3 (10.4)	<0.001*,†
Fasting glucose (mg/dL)	98.7 (0.2)	97.6 (0.8)	98.4 (1.2)	0.41
HDL-cholesterol (mg/dL)				
Men	48.2 (0.2)	47.5 (0.5)	47.1 (0.7)	0.14
Women	56.3 (0.2)	59.3 (1.4)	59.3 (1.8)	0.03
SBP (mmHg)	116.3 (0.2)	116.7 (0.5)	115.8 (0.7)	0.55
DBP (mmHg)	75.7 (0.1)	77.6 (0.4)	77.5 (0.6)	<0.001*,†

Data are presented as weighted percentages (standard errors [SEs]) for categorical variables or weighted means (SEs) for continuous variables, unless otherwise stated.

*P*-values were calculated using a one-way analysis of variance for continuous variables and chi-square test for categorical variables.

Post-hoc analyses with Bonferroni’s correction were performed between *never vs. ever, †never vs. current, and ‡ever vs. current e-cig users. A *P*-value <0.017 was considered significant.

a) Heavy alcohol consumption was defined as the consumption of ≥7 drinks in men and ≥5 drinks in women on an occasion.

b) Adequate physical activity was defined as 1) at least 150–300 minutes of moderate-intensity physical activity per week, 2) 75–150 minutes of vigorous-intensity physical activity per week, or 3) an equivalent combination of moderate- and vigorous-intensity aerobic activities.

WC: waist circumference, HDL: high-density lipoprotein, SBP: systolic blood pressure, DBP: diastolic blood pressure.

The associations between the five MetS components and e-cig use status are shown in Table 2. Current e-cig users showed a significantly higher OR for abdominal obesity and hypertriglyceridemia than never e-cig users (OR: 1.88, 95% CI: 1.41–2.50, *P*_trend_ <0.001 and OR: 1.32, 95% CI 1.00–1.74, *P*_trend_ = 0.01, respectively). These relationships remained significant after adjusting for covariates. Low HDL-cholesterol levels showed a trend for a correlation with e-cig use status; however, the trend was not statistically significant. No significant relationships were observed between high fasting glucose and BP levels and e-cig use status.

**Table 2:** Association between metabolic syndrome components and electronic cigarette exposure.

	Never user (n = 13,459)	Ever user (n = 954)	Current user (n = 325)	*P*_trend_
	Reference	OR (95% CI)	OR (95% CI)	
**Abdominal obesity**				
Model 1	1	1.48 (1.25–1.75)	1.91 (1.44–2.52)	<0.001
Model 2	1	1.45 (1.21–1.74)	1.87 (1.41–2.48)	<0.001
Model 3	1	1.44 (1.20–1.72)	1.89 (1.42–2.51)	<0.001
Model 4	1	1.42 (1.19–1.70)	1.88 (1.41–2.50)	<0.001
**High triglycerides**				
Model 1	1	1.64 (1.41–1.92)	1.80 (1.37–2.36)	<0.001
Model 2	1	1.22 (1.30–1.44)	1.30 (1.00–1.71)	<0.01
Model 3	1	1.20 (1.01–1.42)	1.32 (1.00–1.74)	<0.01
Model 4	1	1.20 (1.10–1.41)	1.32 (1.00–1.74)	0.01
**High fasting glucose**				
Model 1	1	1.03 (0.86–1.24)	1.20 (0.90–1.60)	0.26
Model 2	1	0.90 (0.75–1.09)	1.04 (0.79–1.39)	0.72
Model 3	1	0.89 (0.73–1.07)	1.05 (0.79–1.40)	0.67
Model 4	1	0.89 (0.74–1.08)	1.05 (0.78–1.40)	0.67
**Low HDL-cholesterol**				
Model 1	1	1.35 (1.13–1.61)	1.40 (1.06–1.85)	<0.001
Model 2	1	1.13 (0.94–1.37)	1.15 (0.87–1.54)	0.16
Model 3	1	1.15 (0.95–1.39)	1.14 (0.86–1.52)	0.15
Model 4	1	1.15 (0.95–1.39)	1.14 (0.86–1.52)	0.16
**High blood pressure**				
Model 1	1	1.29 (1.08–1.54)	0.96 (0.73–1.26)	0.13
Model 2	1	1.23 (1.03–1.47)	0.90 (0.68–1.20)	0.49
Model 3	1	1.19 (1.00–1.42)	0.91 (0.68–1.21)	0.59
Model 4	1	1.19 (0.97–1.42)	0.91 (0.68–1.21)	0.60

a) Metabolic syndrome was defined in accordance with the modified Third National Cholesterol Education Program Expert Panel on Detection, Evaluation, and Treatment of High Blood Cholesterol in Adults (NCEP-ATP III) criteria and the abdominal obesity criteria of the Korean Society for the Study of Obesity.

b) Model 1 was adjusted for age and sex; Model 2 additionally adjusted for conventional cigarette exposure; Model 3 was additionally adjusted for alcohol consumption; and Model 4 was additionally adjusted for physical activity, household income, and education level.

c) The *P*-value for trend was measured using a logistic regression analysis considering electronic cigarette exposure as a continuous variable.

OR: odds ratio, CI: confidence interval.

The associations between MetS and e-cig use status are shown in Table 3. The prevalence rates of MetS were 23.6%, 26.8%, and 25.9% in never, ever, and current e-cig users, respectively (*P*_trend_ = 0.08). After adjustment for covariates, the OR for MetS was 1.25 (95% CI: 1.03–1.51) in ever e-cig users and 1.27 (95% CI: 0.96–1.69) in current e-cig users, compared with that in never e-cig users (*P*_trend_ = 0.01). The results of the unweighted analysis of the association between MetS and e-cig use are shown in Table 4. After adjustment for covariates, the OR for MetS was 1.40 (95% CI: 1.08–1.81) in current e-cig users and 1.20 (95% CI: 1.02–1.42) in ever e-cig users compared to that in never e-cig users (*P*_trend_ <0.01).

**Table 3:** Association between metabolic syndrome and electronic cigarette exposure.

	Never user (n = 13,459)	Ever user (n = 954)	Current user (n = 325)	*P*_trend_
	Reference	OR (95% CI)	OR (95% CI)	
Prevalence of MetS, % (SE)	23.6 (0.4)	26.8 (1.6)	25.9 (0.2)	0.08
Model 1	1	1.51 (1.26–1.82)	1.53 (1.16–2.01)	<0.001
Model 2	1	1.28 (1.05–1.54)	1.27 (0.96–1.69)	0.01
Model 3	1	1.26 (1.04–1.52)	1.28 (0.96–1.70)	0.01
Model 4	1	1.25 (1.03–1.51)	1.27 (0.96–1.69)	0.01

a) Model 1 was adjusted for age and sex; Model 2 was additionally adjusted for conventional cigarette exposure; Model 3 was additionally adjusted for alcohol consumption; and Model 4 was additionally adjusted for physical activity, household income, and education level.

b) The *P*-value for trend was measured using a logistic regression analysis considering electronic cigarette exposure as a continuous variable.

OR: odds ratio, CI: confidence interval.

**Table 4:** Unweighted analysis of the association between metabolic syndrome and electronic cigarette exposure.

	Never user (n = 13,459)	Ever user (n = 954)	Current user (n = 325)	*P*_trend_
	Reference	OR (95% CI)	OR (95% CI)	
Model 1	1	1.47 (1.25–1.73)	1.72 (1.33–2.22)	<0.001
Model 2	1	1.22 (1.03–1.44)	1.40 (1.08–1.82)	<0.01
Model 3	1	1.20 (1.01–1.42)	1.40 (1.08–1.82)	<0.01
Model 4	1	1.20 (1.01–1.42)	1.40 (1.08–1.81)	<0.01

a) Model 1 was adjusted for age and sex; Model 2 was additionally adjusted for conventional cigarette exposure; Model 3 was additionally adjusted for alcohol consumption; and Model 4 was additionally adjusted for physical activity, household income, and education level.

b) The *P*-value for trend was measured using a logistic regression analysis considering electronic cigarette exposure as a continuous variable.

OR: odds ratio, CI: confidence interval.

Results of the subgroup analysis conducted among active smokers to determine the association between e-cig use status and MetS are shown in Table 5. Dual use of e-cigs and cigarettes was associated with an increased OR for abdominal obesity. The OR for abdominal obesity in dual users was 1.71 (95% CI: 1.25–2.34) compared with that in never e-cig users.

**Table 5:** Association between metabolic syndrome and electronic cigarette exposure among active smokers (n = 3,278).

	Never user (n = 2,263)	Ever user (n = 774)	Current user (n = 271)	*P*_trend_
	Reference	OR (95% CI)	OR (95% CI)	
Metabolic syndrome	1	1.14 (0.92–1.43)	1.13 (0.82–1.55)	0.25
Abdominal obesity	1	1.28 (1.04–1.58)	1.71 (1.25–2.34)	<0.001
High triglyceride	1	1.11 (0.91–1.36)	1.24 (0.91–1.70)	0.13
High fasting glucose	1	0.92 (0.73–1.15)	1.02 (0.73–1.42)	0.78
Low HDL-cholesterol	1	1.13 (0.90–1.41)	1.13 (0.82–1.55)	0.29
High blood pressure	1	1.15 (0.94–1.41)	0.78 (0.56–1.07)	0.57

a) In all analyses, age, sex, alcohol consumption, physical activity, household income, and education level were adjusted as multivariables.

b) The *P*-value for trend was measured using a logistic regression analysis considering electronic cigarette exposure as a continuous variable.

OR: odds ratio, CI: confidence interval, HDL: high-density lipoprotein.

## Discussion

In the present study, we observed that e-cig use was significantly associated with an increased OR for MetS. The OR for MetS was the highest in current e-cig users and the lowest in never e-cig users. This significant difference might be attributable to the increased ORs for abdominal obesity, high TG levels, and low HDL-cholesterol levels. To the best of our knowledge, this is the first study to evaluate the association between e-cig use and MetS in the general population. These results indicate that e-cigs may play a role in inducing several metabolic abnormalities, and these findings could form a basis for further research on the types, chemical components, and consumption patterns of e-cigs that are responsible for this relationship.

Interestingly and importantly, 85% of current e-cig users continued to use cigarettes. Dual users were found to have a higher OR for abdominal obesity than former smokers, current e-cig users, and never e-cig users. This high proportion of dual use is consistent with the findings from other countries. In the United States, nearly 93% of e-cig users consume cigarettes concurrently [2], as do 83% of e-cig users in France [3], 76% of e-cig users (120 dual users among 159 current e-cig users) in Japan [14], and 74% of e-cig users (174 dual users among 235 current e-cig users) in Germany [15]. In addition, current e-cig users had higher income and education levels than never e-cig users, which is consistent with the findings from a 2016 report by the International Tobacco Survey Group of Korea [16]. Similarly, Simon et al. reported that individuals with a high socio-economic status were more likely to be exposed to e-cig advertisement, and this exposure was associated with high e-cig use among groups with a high socio-economic status [17]. Traditional cigarette users with high educational levels were more likely to switch to e-cig use [18].

The association between cigarette use and metabolic abnormalities has been widely evaluated since Facchini et al. reported the effect of cigarette smoke on insulin resistance in 1992 [19]. They reported that cigarette smoke significantly disrupted lipid homeostasis by increasing TG levels and decreasing HDL-cholesterol levels, which is consistent with the current results. A recent study comparing smokers and non-smokers without diabetes also showed that smoking is associated with higher levels of fasting glucose and insulin resistance [20]. In a study of 3,338 Japanese individuals, current smokers were found to have a 1.75-times higher risk for type 2 diabetes than never smokers [21]. A Korean study summarized data from the 1998 KNHANES and concluded that smoking cigarettes was significantly related to MetS in a dose-dependent manner, after adjusting for age and sex [22]. A meta-analysis, carried out by a Chinese group, including 13 studies and 56,691 individuals demonstrated a dose-dependent relationship between smoking cigarettes and MetS in men [23]. Active cigarette users showed the highest risk for MetS (relative risk [RR]: 1.34, 95% CI: 1.20–1.50), followed by former cigarette users (RR: 1.19, 95% CI: 1.00–1.42). Golli et al. used a rat model to assess the association between e-cig use and metabolic consequences [24]. An intraperitoneally injected e-cig liquid without nicotine altered hepatic function and glucose metabolism in the rat model. The serum aspartate transaminase, alanine transaminase, alkaline phosphatase, and lactate dehydrogenase levels increased after short-term exposure (28 days) to an e-cig liquid without nicotine. The e-cig liquid without nicotine induces GSK3β up-regulation, which inhibits glycogen synthesis. Inhalation of nicotine and e-cig smoke caused a 10–15% increase in oxidative stress and nitroxidative stress [25]. To the best of our knowledge, there has been no direct evidence showing the effect of e-cig use on MetS development, and thus, further longitudinal studies are needed to identify this relationship.

This study showed an association between e-cig use and MetS. However, the underlying mechanisms of this relationship are still not well understood, and the specific e-cig components responsible for these results remain unknown. It is plausible to assume that the negative impacts of e-cig use on metabolism are similar to those of cigarette use as e-cigs also contain varying levels of toxicants such as nicotine, based on the device, as well as additive materials such as flavors and solvent carriers [26]. For example, formaldehyde—a major constituent of e-cigs and a causative factor of oxidative stress—is emitted through the heating process of the e-cig device [27]. As the potential role of oxidative stress in the pathogenesis of MetS is evolving [28], the effects of e-cigs on the imbalance between the systemic activation of reactive oxygen species and the biological detoxification process could be responsible for the observed relationship. Compared with tobacco-naïve individuals, e-cig users showed a disturbed autonomic balance between the predominance of sympathetic tone and increased levels of oxidative stress markers (i.e., low-density lipoprotein oxidizability) [29]. Meanwhile, previous studies have evaluated the negative impact of e-cig use on physiologic changes. A previous *in vitro* model showed that flavoring materials without nicotine induced monocytes to release cytotoxic interleukin-8 and contributed to the production of reactive oxygen species [30], biomarkers well known to reflect oxidative stress and tissue damage [31]. Using human body specimens, Wu et al. reported that exposure to e-cigs without nicotine was associated with inflammation of the airway epithelium [32]. In addition, e-cig extracts induced platelet activation, aggregation, and adhesion in 50 healthy volunteers [33]. As platelet dysfunction and highly thrombotic conditions play important roles in the development of cardiovascular diseases, this result indicates the negative effect of e-cig use on the cardiovascular system. Other studies have further described the impacts of e-cig use on the cardiovascular system [34].

Although our study findings represent a significant advancement in our understanding of the health-related adverse effects of e-cig use, there are several limitations to our study. First, considering the cross-sectional nature of this study, the results should be interpreted with caution as the causal relationships are unclear. Moreover, prospective studies must be conducted on the effects of e-cig use on various metabolic abnormalities in the human body. Second, data on the use of e-cigs and cigarettes were lacking. Although the study findings were adjusted for smoking status as a covariate, data on other potential confounders such as cigarette pack-years (the 2013–2015 KNHANES only assessed pack-years of smoking in current smokers but not in former smokers), types of e-cigs, flavors of e-cigs, consumption pattern, and indoor use of e-cigs were unavailable in the KNHANES.

E-cigs are considered safer than traditional cigarettes; however, being less harmful does not guarantee safety. Although the data from animal studies provide useful insights on the effects of e-cig use, caution is required while interpreting the results of these studies due to the differences in materials and methods, species, and time of exposure. Since an increasing number of people are consuming e-cigs and since many people are dual users, research on health-related concerns is currently in progress. Therefore, efforts are needed to determine the real-world effects of e-cig use on the human body, especially in terms of metabolic abnormalities. In addition, the effect of e-cig use on metabolic outcomes needs to be evaluated according to the flavors and types of e-cigs in future studies.

## Conclusion

E-cig exposure was associated with an increased risk of MetS, and abdominal obesity, low HDL-cholesterol levels, and high TG levels were thought to be the main contributors to this relationship. The dual use of e-cigs and traditional cigarettes showed an increased OR for abdominal obesity. Our results support the hypothesis that e-cig use is associated with MetS and are in line with the results of several previous studies emphasizing that e-cigs are not as safe as previously believed. Further studies are needed to clarify the underlying mechanisms contributing to these findings.
